# Regional differences in rates of HIV-1 viral load monitoring in Canada: Insights and implications for antiretroviral care in high income countries

**DOI:** 10.1186/1471-2334-10-40

**Published:** 2010-02-25

**Authors:** Janet M Raboud, Mona R Loutfy, DeSheng Su, Ahmed M Bayoumi, Marina B Klein, Curtis Cooper, Nima Machouf, Sean Rourke, Sharon Walmsley, Anita Rachlis, P Richard Harrigan, Marek Smieja, Christos Tsoukas, Julio SG Montaner, Robert S Hogg, the CANOC Collaboration

**Affiliations:** 1 Dalla Lana School of Public Health, University of Toronto, Toronto, Canada; 2 Division of Infectious Disease, University Health Network, Toronto, Canada; 3 Department of Medicine, University of Toronto, Toronto, Canada; 4 Women's College Research Institute, Women's College Hospital, Toronto, Canada; 5 Department of Health Policy, Management and Evaluation, University of Toronto, Toronto, Canada; 6 Maple Leaf Medical Clinic, Toronto, Canada; 7 Centre for Research on Inner City Health, The Keenan Research Centre in the Li Ka Shing Knowledge Institute, St. Michael's Hospital, Toronto, Canada; 8 Division of General Internal Medicine, St. Michael's Hospital, Toronto, Canada; 9 Department of Medicine, McGill University Health Centre, Montreal, Canada; 10 University of Ottawa, Ottawa, Canada; 11 Clinique médicale l'Actuel, Montreal, Canada; 12 Department of Psychiatry, University of Toronto, Toronto, Canada; 13 Ontario HIV Treatment Network, Toronto, Canada; 14 Division of Infectious Disease, Sunnybrook Health Sciences Centre, Toronto, Canada; 15 Faculty of Medicine, University of British Columbia, Vancouver, Canada; 16 The British Columbia Centre for Excellence in HIV/AIDS, Vancouver, Canada; 17 Department of Pathology and Molecular Medicine, McMaster University, Hamilton, Canada; 18 Faculty of Health Sciences, Simon Fraser University, Vancouver, Canada

## Abstract

**Background:**

Viral load (VL) monitoring is an essential component of the care of HIV positive individuals. Rates of VL monitoring have been shown to vary by HIV risk factor and clinical characteristics. The objective of this study was to determine whether there are differences among regions in Canada in the rates of VL testing of HIV-positive individuals on combination antiretroviral therapy (cART), where the testing is available without financial barriers under the coverage of provincial health insurance programs.

**Methods:**

The Canadian Observational Cohort (CANOC) is a collaboration of nine Canadian cohorts of HIV-positive individuals who initiated cART after January 1, 2000. The study included participants with at least one year of follow-up. Generalized Estimating Equation (GEE) regression models were used to determine the effect of geographic region on (1) the occurrence of an interval of 9 months or more between two consecutive recorded VL tests and (2) the number of days between VL tests, after adjusting for demographic and clinical covariates. Overall and regional annual rates of VL testing were also reported.

**Results:**

3,648 individuals were included in the analysis with a median follow-up of 42.9 months and a median of 15 VL tests. In multivariable GEE logistic regression models, gaps in VL testing >9 months were more likely in Quebec (Odds Ratio (OR) = 1.72, p < 0.0001) and Ontario (OR = 1.78, p < 0.0001) than in British Columbia and among injection drug users (OR = 1.68, p < 0.0001) and were less likely among older individuals (OR = 0.77 per 10 years, p < 0.0001), among men having sex with men (OR = 0.62, p < 0.0001), within the first year of cART (OR = 0.15, p < 0.0001), among individuals on cART at the time of the blood draw (OR = 0.34, p < 0.0001) and among individuals with VL < 50 copies/ml at the previous visit (OR = 0.56, p < .0001).

**Conclusions:**

Significant variation in rates of VL testing and the probability of a significant gap in testing were related to geographic region, HIV risk factor, age, year of cART initiation, type of cART regimen, being in the first year of cART, AIDS-defining illness and whether or not the previous VL was below the limit of detection.

## Background

Viral load (VL) testing is an essential component of the care of HIV-positive individuals, both with regard to timing of initiation of antiretroviral therapy (ART) and to monitoring of virologic response to combination ART (cART) [1]. The goal of cART is sustained virologic suppression, defined as a VL below the level of detection of the test performed [1]. Guidelines recommend that HIV-positive individuals receive VL testing at intervals of three to four months as standard of care [1]. CD4 count monitoring is important for deciding when to start cART and for determining prognosis, but alone is insufficient as a marker of treatment efficacy as it does not identify individuals experiencing virologic rebound or failure [2]. Early determination of virologic rebound and failure is one of the most crucial components of HIV management as it contributes to the reduction of ART drug resistance [3]. Lastly, VL monitoring has also been shown to promote treatment adherence, which is additionally important for maintaining virologic suppression and reducing the evolution of drug resistance [4].

Access to VL testing has been studied previously. In an Ontario cohort, injection drug use, younger age and residence in Toronto were associated with lower VL testing rates [5]. In another study, drug users were also found to be at risk for irregular VL monitoring [6]. In a study of individuals who initiated ART between 1994 and 2000, individuals with low CD4 counts and high VLs had the highest rates of laboratory testing [7].

In this study, we examine whether there are regional differences in patterns of VL testing among individuals who initiated cART therapy since January 1, 2000 in Canada, where VL testing is available without charge to all HIV-positive residents as part of the provincial universal health insurance plans. Furthermore, we identified demographic and clinical factors associated with suboptimal frequency of VL testing.

## Methods

The Canadian Observational Cohort (CANOC) collaboration is a Canadian cohort study of antiretroviral naïve HIV-positive patients initiating cART since January 1^st ^2000. The study was established in March 2008 with funding from the Canadian Institutes of Health Research (grant# 711098) and the CIHR Canadian HIV Trials Network (CTN242) and includes cohorts and investigators from across the country (listed at the end of the manuscript). The collaboration is open to all Canadian HIV treatment cohorts with more than 100 eligible patients.

### Participating cohorts

Data used in this analysis were from nine cohorts of HIV-positive individuals in British Columbia (BC), Ontario, and Quebec, including the BC Centre for Excellence in HIV/AIDS Drug Treatment Program, Montreal Chest Institute Immunodeficiency Cohort, The Electronic Antiretroviral Therapy, Clinique Médicale l'Actuel, The Canadian HIV/HCV Co-infection Cohort, Ontario Cohort Study, Maple Leaf Medical Clinic, Toronto General Hospital and Ottawa Hospital HIV/HCV Cohort [8].

Patient selection and data extraction were performed at the data centres of the participating cohort sites. In provinces with multiple cohorts, VL data were entered from each cohort site and not from a provincial data source. Non-nominal data from each cohort on a predefined set of demographic, laboratory, and clinical variables were then pooled and analyzed at the Project Data Centre in Vancouver. All participating cohorts have received approval from their institutional ethics boards and governance committees to contribute non-nominal patient-specific data to CANOC. Ownership of individual cohort data remains with the contributing cohort and cohort data can only be used for studies approved by the CANOC Steering Committee.

### Eligibility Criteria

Eligibility criteria for inclusion in CANOC were documented HIV infection, residence in Canada, aged 18 years and over, initiation of three or more antiretroviral drugs for the first time (i.e. ART-naïve cART start) after January 1, 2000, and a viral load measurement and CD4 cell count within 6 months of the start of therapy. To be included in this analysis, individuals had to have at least one year of follow-up. Viral load measurements were available both before and after starting cART.

### Statistical Methods

Three measures were used to assess the frequency of VL testing. First, the primary outcome of interest was defined as a gap between VL tests in excess of 9 months, corresponding to at least three missed or delayed tests assuming the optimal frequency between tests is three months. This was felt to be a clinically important gap in VL testing. Second, the annual rate of VL testing was calculated by dividing the total number of tests for an individual by the duration of follow-up for that individual in years. Third, the time interval, defined as the number of days between two successive VL tests for a subject, was examined.

Demographic and clinical characteristics such as gender, race, HIV risk factors, age, geographic region, CD4 count and type of cART regimen were compared among regions with chi square tests for categorical variables and Wilcoxon rank sum tests for continuous variables.

The proportion of individuals with at least one suboptimal interval and the annual rate of VL testing experienced were compared among regions and by demographic and clinical characteristics with the chi square test and Wilcoxon rank sum test respectively. Generalized Estimating Equation (GEE) logistic regression models, which account for correlation among multiple observations within subjects, were used to determine factors associated with the occurrence of a suboptimal testing interval[9]. An exchangeable correlation structure was assumed for this model. As a form of sensitivity analysis, we repeated the analysis by defining a suboptimal interval as six months or more, corresponding to at least two missed or delayed tests if the optimal frequency between tests is three months.

The relationships between the length of the VL inter-test interval and individual characteristics were examined using GEE linear regression models. Plasma VL levels, CD4 counts, characteristics of the antiretroviral regimen and the calendar year of VL tests were treated as time-varying covariates in all regression models. Covariates with a p < 0.10 in the univariate regression models were considered as candidates for inclusion in the multivariable model.

### Data cleaning

When there was a gap between two consecutive VL tests in excess of nine months, the accompanying CD4 test dates were examined to validate the VL test dates. Viral load tests within 8 days of each other and with results within 0.1 log_10 _copies/mL were considered to be duplicate measurements. In such cases, the first VL date was kept and the average of the VL measurements was assigned as the VL value.

## Results

### Study cohort and overall VL testing

3,648 subjects met cohort inclusion criteria. Demographic characteristics are described by region in Table 1. The median number of VL tests and the median length of follow-up were 15 (interquartile range (IQR) [9-13][14-22]) and 42.9 months (IQR [25.4-64.4]), respectively. The median rate of testing was 4.3 VL measurements per year (IQR [3.4-5.5]). On average, 83% of the population had more than 3 tests per year and 58% had more than 4 tests per year. Of 57,308 intervals between VL tests, 2.3% were > 9 months and 6.8% were > 6 months. The median annual rates of VL testing were 4.9 (IQR [3.8-6.3]) in BC, 3.9 (IQR [3.1-4.8]) in Ontario and 4.0 (IQR [3.2-4.8]) in Quebec.

**Table 1:** Comparison of Baseline Demographic and Clinical Characteristics by Region

		*Region*			
			
*Characteristics*	*Total (n = 3648)*	*BC (n = 1674)*	*Ontario (n = 1143)*	*Quebec (n = 831)*	*p value*
Age	40 (34-46)	40 (34-47)	39 (34-45)	40 (34-46)	< 0.01
Male	2925 (80%)	1328 (79%)	934 (82%)	663 (80%)	0.28
Race†					
Caucasian	956 (52%)	552 (45%)	309 (50%)	95 (84%)	< 0.0001
Black	181 (10%)	31 (3%)	138 (26%)	12 (11%)	
First Nation	121 (7%)	103 (9%)	15 (3%)	3 (3%)	
Mixed	465 (25%)	464 (38%)	1 (<%	0 (0%)	
Other	128 (7%)	66 (5%)	59 (11%)	3 (3%)	
Missing	1837	497	622	718	
Risk factor‡					
MSM	1015 (34%)	203 (12%)	362 (66%)	450 (60%)	< 0.0001
IDU	632 (21%)	442 (26%)	82 (13%)	108 (14%)	< 0.0001
Heterosexual	486 (16%)	98 (6%)	188 (35%)	200 (27%)	< 0.0001
Endemic country	233 (19%)	0 (0%)	92 (20%)	141 (19%)	0.52
Blood product recipient	43 (1%)	10 (1%)	25 (6%)	8 (1%)	< 0.0001
Other/Missing	1554 (43%)	961 (57%)	569 (45%)	147 (9%)	< 0.0001
AIDS defining illness	481 (13%)	259 (15%)	115 (10%)	107 (13%)	< 0.001
cART initiation date	2003 (2001-05)	2003 (2001-05)	2004 (2002 - 05)	2004 (2002-05)	< 0.0001
Type of cART					
NNRTI-based	1586 (43%)	753 (45%)	512 (45%)	321 (39%)	< 0.0001
Boosted PI-based	1433 (39%)	726 (43%)	397 (35%)	310 (37%)	
Single PI-based	384 (11%)	151 (9%)	137 (12%)	96 (12%)	
Other cART	245 (7%)	44 (3%)	97 (8%)	104 (13%)	
CD4 count (cells/mm^3^)	190 (100-277)	170 (80-260)	202 (107-296)	208 (127-290)	< 0.0001
CD4 count category					
< 200 cells/mm^3^	1921 (53%)	981 (59%)	555 (49%)	385 (47%)	< 0.0001
200 - 350 cells/mm^3^	1175 (32%)	486 (29%)	374 (33%)	315 (38%)	
> 350 cells/mm^3^	544 (15%)	207 (12%)	212 (19%)	125 (15%)	
VL (log_10 _copies/mL)	4.9 (4.4-5.1)	5.0 (4.6-5.0)	4.8 (4.3-5.3)	4.8 (4.2-5.2)	< 0.01
VL <50 copies/mL	139 (4%)	56 (3%)	48 (4%)	35 (4%)	0.40
Hepatitis C co-infection	736 (30%)	501 (44%)	124 (22%)	111 (15%)	< 0.0001

MSM = men who have sex with men, IDU = injection drug user, cART = combination antiretroviral therapy, NNRTI = non nucleoside reverse transcriptase inhibitor, PI = protease inhibitor, VL = viral load.

†Race percentages are calculated as percentages of non-missing values. ‡Risk factors are not mutually exclusive. Results are N (%) or median (interquartile range). Missing values are only reported for variables with more than 25% missing data.

### Analysis of the annual rate of VL testing

In univariate analyses, higher annual rates of VL testing were associated with residence in BC, age > 40 years, white race, male gender, later year of cART initiation, pretreatment VL ≥ 10^5 ^copies/mL, an HIV risk category of "men who have sex with men" (MSM), history of an AIDS-defining illness, boosted-protease inhibitor (PI)-based cART regimen, not being co-infected with Hepatitis C and not being an injection drug user (IDU) (Table 2).

**Table 2:** Annual rates of viral load testing and proportions of participants with gaps in testing by baseline demographic and clinical characteristics

		*annual rate of VL testing*		*Subjects having ≥ 1 gap of >9 months*		*Subjects having ≥ 1 gap of >6 months*	
*Characteristics*	*N*	*median (IQR)*	*p value*	*# (%)*	*p value*	*# (%)*	*p value*
Overall	3648	4.3 (3.4-5.5)		943 (26%)		1848 (51%)	
Region							
BC	1674	4.9 (3.8-6.3)	< .0001	347 (21%)	< .0001	672 (40%)	< .0001
Ontario	1143	3.9 (3.1-4.8)		357 (31%)		699 (61%)	
Quebec	831	4.0 (3.2-4.8)		239 (29%)		477 (57%)	
Age (years)							
≤ 40	1942	4.1 (3.2-5.3)	< .0001	614 (32%)	< .0001	1122 (58%)	< .0001
> 40	1706	4.5 (3.6-5.8)		329 (19%)		726 (43%)	
Gender							
Female	723	3.9 (3.1-5.0)	< .0001	255 (35%)	< .0001	475 (66%)	< .0001
Male	2925	4.4 (3.5-5.7)		688 (24%)		1373 (47%)	
Caucasian							
No	855	4.4 (3.4-5.7)	0.33	255 (30%)	0.04	463 (54%)	< .001
Yes	956	4.4 (3.4-5.9)		244 (26%)		439 (46%)	
Risk Factor							
IDU	632	4.0 (3.0-5.1)	< .0001	247 (39%)	< .0001	417 (66%)	< .0001
MSM	976	4.3 (3.5-5.4)		186 (19%)		421 (43%)	
Blood product recipient	32	4.1 (3.5-5.1)		8 (25%)		18 (56%)	
Endemic	209	4.1 (3.4-4.8)		60 (29%)		128 (61%)	
Heterosexual contact	245	4.2 (3.4-5.7)		64 (26%)		135 (55%)	
Not reported/Other	1554	4.5 (3.4-5.8)		378 (24%)		729 (47%)	
AIDS defining illness							
No	3167	4.2 (3.3-5.5)	< .0001	858 (27%)	< .0001	1641 (52%)	< .001
Yes	481	4.7 (3.8-5.9)		85 (18%)		207 (43%)	
Type of cART							
NNRTI-based	1586	4.1 (3.2-5.0)	< .0001	425 (27%)	< .0001	874 (55%)	< .0001
Boosted PI-based	1433	5.0 (3.7-6.5)		292 (20%)		573 (40%)	
Single PI-based	384	4.0 (3.1-4.9)		149 (39%)		256 (67%)	
Other cART	245	3.9 (3.2-4.7)		77 (31%)		145 (59%)	
Year of initiating cART							
2000	482	3.7 (2.8-4.7)	< .0001	219 (45%)	< .0001	346 (72%)	< .0001
2001-2004	2036	4.2 (3.3-5.3)		599 (29%)		1132 (56%)	
>2004	1130	4.9 (3.9-6.4)		125 (11%)		370 (33%)	
CD4 count							
< 200 cells/mm^3^	1921	4.4 (3.4-5.7)	< .0001	474 (25%)	< .001	941 (49%)	< .0001
200 - 350 cells/mm^3^	1175	4.3 (3.4-5.5)		286 (24%)		560 (48%)	
> 350 cells/mm^3^	544	4.1 (3.0-5.2)		181 (33%)		341 (63%)	
VL (copies/mL)							
≥ 50	3509	4.3 (3.4-5.6)	< .001	905 (26%)	0.68	1770 (50%)	0.19
< 50	139	3.9 (2.9-4.8)		38 (27%)		78 (56%)	
Hepatitis C Co-infection							
No	1712	4.4 (3.6-5.6)	< .0001	346 (20%)	< .0001	756 (44%)	< .0001
Yes	736	4.1 (3.0-5.3)		281 (38%)		473 (64%)	

MSM = men who have sex with men, IDU = injection drug use, cART = combination antiretroviral therapy, NNRTI = non nucleoside reverse transcriptase inhibitor, PI = protease inhibitor.

† Risk factors were grouped hierarchically for comparison purposes: IDU, MSM, blood product recipient, origin/residence in an HIV-endemic area, heterosexual transmission

### Analysis of gaps of greater than nine months and six months

Of the 3,648 patients eligible for the analysis, 26% and 51% of the population had experienced at least one nine-month and one six-month gap during their follow-up, respectively. Proportions of patients with at least one nine-month gap and with at least one six-month gap are shown by demographic and clinical characteristics in Table 2. Results of univariate GEE logistic regression models are shown in Table 3. In the multivariable GEE logistic regression model (Table 4), gaps of both nine and six months were significantly more likely to occur in Ontario and Quebec and among IDUs. Gaps of both nine and six months were significantly less likely to occur among older individuals, among MSM, in recent calendar years of VL test, among individuals on cART, in the first year of cART and if the VL had been suppressed at the previous visit.

**Table 3:** Univariate GEE regression models of interval (days), probability of >9 and >6 months between viral load measurements

	*Interval (days)*		*Gaps of >9 months*		*Gaps of >6 months*	
*Covariates*	*Estimate (95% CI)*	*p value*	*OR (95% CI)*	*p value*	*OR (95% CI)*	*p value*
Region						
Quebec	24.1 (20.2, 28.0)	< .0001	1.65 (1.37, 1.98)	< .0001	1.71 (1.50, 1.96)	< .0001
Ontario	25.6 (22.2, 28.9)	< .0001	1.77 (1.51, 2.08)	< .0001	2.14 (1.90, 2.41)	< .0001
BC (reference)	0		1		1	
Age (per 10 years)	-7.0 (-8.6, -5.3)	< .0001	0.70 (0.65, 0.76)	< .0001	0.76 (0.72, 0.81)	< .0001
Male	-12.9 (-16.9, -8.8)	< .0001	0.58 (0.50, 0.68)	< .0001	0.62 (0.55, 0.69)	< .0001
Caucasian	-2.1 (-6.6, 2.4)	0.36	0.78 (0.64, 0.95)	0.01	0.83 (0.71, 0.97)	0.02
Risk factor						
MSM	2.2 (-1.1, 5.6)	0.19	0.74 (0.61, 0.89)	< .01	0.86 (0.75, 0.97)	0.02
IDU	18.5 (13.1, 23.9)	< .0001	2.06 (1.74, 2.44)	< .0001	1.80 (1.58, 2.04)	< .0001
Heterosexual	14.3 (9.4, 19.3)	< .0001	1.55 (1.26, 1.89)	< .0001	1.57 (1.36, 1.81)	< .0001
Endemic country	-0.1 (-6.3, 6.1)	0.98	1.05 (0.79, 1.39)	0.74	1.08 (0.89, 1.31)	0.44
Blood products	6.3 (-3.8, 16.4)	0.22	0.93 (0.52, 1.65)	0.79	1.19 (0.76, 1.89)	0.45
Year of initiating cART						
>2004	-31.0 (-37.4, -24.6)	< .0001	0.36 (0.28, 0.46)	< .0001	0.49 (0.41, 0.57)	< .0001
2001-2004	-18.4 (-24.7, -12.1)	< .0001	0.62 (0.52, 0.73)	< .0001	0.70 (0.61, 0.80)	< .0001
2000 (ref.)	0		1		1	
1^st ^year of cART	-36.1 (-37.6, -34.6)	< .0001	0.15 (0.13, 0.18)	< .0001	0.27 (0.25, 0.30)	< .0001
Type of cART						
NNRTI-based	0		1		1	
Boosted PI-based	-16.7 (-20.0, -13.4)	< .0001	0.76 (0.64, 0.89)	< .001	0.71 (0.63, 0.80)	< .0001
Single PI-based	5.4 (-0.3, 11.0)	0.06	1.37 (1.12, 1.68)	< .01	1.19 (1.02, 1.40)	0.03
Other cART	6.2 (-0.2, 12.6)	0.06	1.17 (0.89, 1.53)	0.26	1.05 (0.86, 1.27)	0.65
Boosted PI (Y/N)	-18.3(-21.4, -15.2)	< .0001	0.70 (0.60, 0.81)	< .0001	0.68 (0.61, 0.76)	< .0001
On cART at current visit	-32.2 (-34.9, -29.6)	< .0001	0.31 (0.28, 0.35)	< .0001	0.38 (0.35, 0.42)	< .0001
On cART at previous visit	-12.4 (-14.5, -10.3)	< .0001	0.58 (0.52, 0.65)	< .0001	0.65 (0.61, 0.70)	< .0001
Baseline CD4 count						
>350 cells/mm^3^	12.7 (7.8, 17.7)	< .0001	1.43 (1.19, 1.72)	< .001	1.51 (1.31, 1.74)	< .0001
200-350 cells/mm^3^	2.7 (-0.7, 6.1)	0.12	0.94 (0.80, 1.10)	0.44	0.97 (0.86, 1.09)	0.57
< 200 cells/mm^3 ^(ref.)	0		1		1	
Baseline VL < 50 copies/mL	17.3 (7.2, 27.3)	< .001	1.17 (0.82, 1.67)	0.38	1.47 (1.13, 1.90)	< .01
Baseline VL (log_10 _copies/mL)	-7.2 (-9.2, -5.3)	< .0001	0.85 (0.79, 0.91)	< .0001	0.84 (0.79, 0.88)	< .0001
VL <50 copies/mL at previous test	18.3 (16.5, 20.0)	< .0001	0.71 (0.64, 0.78)	< .0001	1.18 (1.11, 1.26)	< .0001
VL (log_10 _copies/mL) at previous visit	-3.1 (-3.9, -2.3)	< .0001	1.23 (1.18, 1.28)	< .0001	1.02 (0.99, 1.04)	0.24
AIDS defining illness	-14.1 (-17.5, -10.6)	< .0001	0.53 (0.42, 0.68)	< .0001	0.59 (0.50, 0.70)	< .0001
Hepatitis C co-infection	17.9 (13.0, 22.7)	< .0001	2.45 (2.06, 2.91)	< .0001	1.98 (1.74, 2.26)	< .0001
Positive for HCV antibodies	14.0 (9.3, 18.7)	< .0001	2.13 (1.81, 2.50)	< .0001	1.64 (1.45, 1.85)	< .0001

MSM = men who have sex with men, IDU = injection drug use, cART = combination antiretroviral therapy, NNRTI = non nucleoside reverse transcriptase inhibitor, PI = protease inhibitor, VL = viral load, HCV = hepatitis C virus

**Table 4:** Multivariate GEE regression models of testing intervals and of probability of >9 and >6 months between viral load measurements

	*Interval (days) between successive tests*		*Probability of an interval >9 months*		*Probability of an interval >6 months*	
*Covariates*	*Estimate (95% CI)*	*p value*	*Odds Ratio (95% CI)*	*p value*	*Odds Ratio (95% CI)*	*p value*
Intercept	125.8 (118.8, 132.9)	< .0001	0.09 (0.07, 0.11)	< .0001	0.20 (0.16, 0.23)	< .0001
Region						
Quebec	20.3 (15.5, 25.0)	< .0001	1.72 (1.39, 2.14)	< .0001	1.61 (1.37, 1.89)	< .0001
Ontario	18.7 (14.3, 23.0)	< .0001	1.78 (1.37, 2.31)	< .0001	1.77 (1.47, 2.14)	< .0001
BC	0		1		1	
Age (per 10 years)	-3.8 (-5.4, -2.2)	< .0001	0.77 (0.70, 0.85)	< .0001	0.77 (0.72, 0.83)	< .0001
Risk factor						
MSM	-9.2 (-13.1, -5.4)	< .0001	0.62 (0.49, 0.78)	< .0001	0.66 (0.56, 0.77)	< .0001
IDU	16.0 (11.1, 20.9)	< .0001	1.68 (1.38, 2.05)	< .0001	1.71 (1.48, 1.99)	< .0001
Year of initiating cART						
>2004	-15.8 (-21.9, -9.7)	< .0001	0.62 (0.46, 0.83)	0.001	0.74 (0.60, 0.91)	0.005
2001-2004	-12.4 (-18.2, -6.6)	< .0001	0.70 (0.57, 0.86)	0.0006	0.75 (0.64, 0.89)	0.0007
2000 (ref.)	0		1		1	
1^st ^year of cART	-24.3 (-26.0, -22.6)	< .0001	0.15 (0.12, 0.20)	< .0001	0.32 (0.29, 0.36)	< .0001
On cART at current visit	-27.8 (-31.0, -24.6)	< .0001	0.34 (0.29, 0.40)	< .0001	0.40 (0.36, 0.45)	< .0001
VL < 50 copies/mL at previous test	8.6 (6.6, 10.7)	< .0001	0.56 (0.48, 0.65)	< .0001	0.90 (0.82, 0.98)	0.02
Boosted PI based cART	-9.1 (-12.2, -6.0)	< .0001			0.82 (0.71, 0.94)	0.006
AIDS defining illness	-4.9 (-8.5, -1.4)	0.007				

MSM = men who have sex with men, IDU = injection drug use, cART = combination antiretroviral therapy, VL = viral load, PI = protease inhibitor,.

### Analysis of the time interval between successive tests

In univariate GEE linear regression models, covariates which were associated with a decrease in the number of days between VL tests included age, male gender, later year of initiating cART, being within the first year of cART, receiving PI-boosted cART regimen, higher baseline VL and having been diagnosed with an AIDS-defining illness (Table 3). Covariates which were associated with an increase in the number of days between VL tests included living in Quebec or Ontario compared to living in BC, not being on cART, having more drugs in the cART regimen, higher baseline CD4 count, being an IDU and having a suppressed VL at the previous test. The predicted time interval between successive tests was 85 days for a 40 year old patient living in BC who started cART between 2002 and 2004, had completed the first year of a boosted-PI based cART regimen, with the most recent VL below the limit of detection, being neither a MSM nor an IDU, and taking cART at the current VL test (Table 4). The predicted time intervals would be 104 or 105 days for a similar patient in Ontario or Quebec, respectively.

## Discussion

There was considerable regional variation in annual rates of VL measurement documented in this Canada-wide study. This variation remained significant even after adjusting for demographic variables such as age and HIV risk factors and clinical variables such as year of cART initiation, type of cART regimen, being in the first year of cART, AIDS-defining illness and whether or not the previous VL was below the limit of detection. In Ontario and Quebec, VL was measured quarterly on average. In BC, rates of VL measurement were even more frequent than guidelines suggest, with an average measurement frequency of almost five times annually.

There are a number of possible explanations for the regional differences in rates of viral load measurement. Some of the difference in measurement rate was due to regional differences in demographic factors such as the proportions of IDUs, who typically have less frequent viral load testing, or pregnant women, in whom viral load is monitored more closely. Further differences could be due to variation in rates of VL blips among regions, after which a repeat VL measurement is typically ordered. Recent data has documented differing rates of blips by VL assay [10,11] and this may explain the higher rates of testing in BC. Regional differences in VL testing policies may also have an impact. While there are no differences among provinces in the official guidelines for the frequency of VL measurement, it is possible that there are differences in the implementation of the guidelines. In Ontario, a VL will not be performed by the laboratory if one has been done within the last 14 days. Furthermore, rates of VL measurement may be higher in British Columbia due to the fact that all antiretroviral drug distribution and VL testing is coordinated through a single center in the province. Lastly, differences in participation rates among provinces in research studies, which may require more frequent VL testing may explain some of regional variation.

Effective therapy should result in at least a 90% or 10-fold (1.0 log_10 _copies/mL) decrease in plasma VL in the first month and suppression to below 50 copies/mL by 24 weeks, depending on the pretreatment VL level [1]. Current guidelines suggest once VL suppression to below 50 copies/mL is confirmed, it should be assessed at regular intervals (e.g. every 3 or 4 months). Isolated episodes of low-level viremia ("blips") are not necessarily predictive of subsequent virologic failure, but consistent elevations of VL above 50 copies/mL meet a strict definition of virologic failure. Emergence of a detectable VL in a previously suppressed patient (i.e. previously consistently < 50 copies/mL) mandates re-evaluation of the case, including repeated testing to confirm whether this represents a "blip" or virologic failure. Confirmed VL rebound should prompt a careful evaluation of regimen tolerability, drug-drug interactions, resistance and patient adherence. CD4+ cell counts should generally be assessed in concert with VL.

In terms of the demographic findings, our findings are similar to those of the study conducted in Ontario several years ago and could likely be generalizable to other settings with universal health care programs. In settings with user-pay programs for virologic monitoring, it seems likely that socioeconomic factors will have a considerably greater influence on rates of testing. In resource limited settings, availability and travel distances are likely to remain significant barriers to regular monitoring of VL levels.

Our results have important implications for guidelines and for research into the monitoring of VL. For patients for whom VL is measured less frequently, virologic rebound will be detected later on average and patients will remain on failing regimens longer than is necessary. In some circumstances, however, it is safe to measure VL less frequently [12]. A reduced monitoring schedule would result in cost savings due to both the cost of the laboratory testing and the cost of the physician time. As ART is a life-long commitment, cost considerations are not insignificant. Further, less frequent VL monitoring would reduce the inconvenience to the patient, which may improve overall compliance. However, while it may be safe and cost effective to extend the period between VL measurements from three to six months in patients with virologic suppression, periods in excess of nine months are less easily justified and place patients at unnecessary risk. Steps should be taken to facilitate more careful VL monitoring in groups at high risk for gaps in testing.

A strength of our study is that VL monitoring is provided free of charge to all HIV-positive individuals in Canada, so that we were able to examine correlates of rates of VL testing in the absence of financial barriers to testing. Further, by combining data from nine sites across Canada, we achieved a large sample size as well as a good representation by region, gender, race, and other demographic characteristics. CANOC represents about a quarter of all HIV-positive people in the country who are currently on ART and approximately half of those who have initiated on more modern regimens since 2000 [13]. This is the largest sample of HIV-positive people on ART compiled in Canada and represents one of the most representative samples put together in a high-income country. One limitation of this dataset is that this information is not currently available on a nation-wide basis, so regional differences in provinces other than BC, Ontario and Quebec could not be examined. A further limitation is that data were missing on race and HIV risk factors for many patients.

Future plans for investigation include further examination of the reasons for the regional differences in rates of VL testing. Also, an economic analysis to determine if the less frequent testing found in Ontario and Quebec is adequate in terms of clinical outcomes and could result in financial savings would be informative. Particularly, an analysis of VL testing frequency in patients with full virologic suppression where less testing may be adequate would be of interest and its impact on cost savings could be important.

## Conclusions

In our setting, with universal health care and similar regional guidelines for viral load testing, significant variation in rates of VL measurement and the probability of a significant gap in testing were related to geographic region, age, HIV risk factors and clinical variables such as year of cART initiation, type of cART regimen, being in the first year of cART, AIDS-defining illness and whether or not the previous VL was below the limit of detection.

## List of abbreviations

AIDS: acquired immune deficiency syndrome; ART: antiretroviral therapy; BC: British Columbia; CANOC: Canadian Observational Cohort; cART: combination antiretroviral therapy; CI: confidence interval; GEE: generalized estimating equation; HCV: hepatitis C virus; HIV: human immunodeficiency virus; IDU: injection drug user; IQR: interquartile range; MSM: men who have sex with men; NNRTI: non nucleoside reverse transcriptase inhibitor; NRTI: nucleoside reverse transcriptase inhibitor; OR: odds ratio; PI: protease inhibitor; VL: viral load.

## Competing interests

The authors declare that they have no competing interests.

## Authors' contributions

DS conducted the statistical analyses under the direction of JMR, MRL and AMB. JMR and MRL drafted the manuscript. RSH, CC, MBK, MRL, NM, JSGM, JMR, SBR, CT are principal investigators of CANOC and contributed cohort data to CANOC. All authors reviewed the manuscript during preparation, provided critical feedback and approved the final manuscript.

## Pre-publication history

The pre-publication history for this paper can be accessed here:

http://www.biomedcentral.com/1471-2334/10/40/prepub
